# Cyclic Tensile Strain Induces Tenogenic Differentiation of Tendon-Derived Stem Cells in Bioreactor Culture

**DOI:** 10.1155/2015/790804

**Published:** 2015-07-01

**Authors:** Yuan Xu, Qiang Wang, Yudong Li, Yibo Gan, Pei Li, Songtao Li, Yue Zhou, Qiang Zhou

**Affiliations:** ^1^Department of Orthopedics, Xinqiao Hospital, Third Military Medical University, Chongqing 400037, China; ^2^Department of Orthopedics, Southwest Hospital, Third Military Medical University, Chongqing 400038, China; ^3^Institute of Pathology and Southwest Cancer Center, Southwest Hospital, Third Military Medical University, Chongqing 400038, China

## Abstract

Different loading regimens of cyclic tensile strain impose different effects on cell proliferation and tenogenic differentiation of TDSCs in three-dimensional (3D) culture in vitro, which has been little reported in previous literatures. In this study we assessed the efficacy of TDSCs in a poly(L-lactide-co-*ε*-caprolactone)/collagen (P(LLA-CL)/Col) scaffold under mechanical stimulation in the custom-designed 3D tensile bioreactor, which revealed that cyclic tensile strain with different frequencies (0.3 Hz, 0.5 Hz, and 1.0 Hz) and amplitudes (2%, 4%, and 8%) had no influence on TDSC viability, while it had different effects on the proliferation and the expression of type I collagen, tenascin-C, tenomodulin, and scleraxis of TDSCs, which was most obvious at 0.5 Hz frequency with the same amplitude and at 4% amplitude with the same frequency. Moreover, signaling pathway from microarray analysis revealed that reduced extracellular matrix (ECM) receptor interaction signaling initiated the tendon genius switch. Cyclic tensile strain highly upregulated genes encoding regulators of NPM1 and COPS5 transcriptional activities as well as MYC related transcriptional factors, which contributed to cell proliferation and differentiation. In particular, the transcriptome analysis provided certain new insights on the molecular and signaling networks for TDSCs loaded in these conditions.

## 1. Introduction

Tendon-derived stem cells (TDSCs) are a potential source of material for the generation of tissue-engineered tendons and repair of injured tendons [1, 2]. TDSCs can differentiate into tenocytes under a variety of stimulation conditions. In vitro, the release of platelet-rich plasma [3] as well as connective tissue growth factor and ascorbic acid [4] can promote TDSCs differentiation into tenocytes. Isodirectional nanofiber scaffolds can induce the above differentiation through integrin- and myosin-mediated mechanical stress pathways [5]. In vivo, tendon tissue regeneration and functional improvement have been observed after the transplantation of TDSCs in a rat patellar tendon window defect model [6].

The function of tendons is to transmit the load between muscles and bones, so tendons withstand sustained mechanical load. Tensile strain is an important part of the environment for tendon/ligament tissues in vivo and can promote the formation of tendon/ligament extracellular matrix (ECM) [7]. TDSCs are natural cells existing in the tendons [8]. The results of recent studies showed that TDSCs were very sensitive to mechanical load [9–11]. After mice were placed on a treadmill for running exercise, the proliferation rate of their TDSCs was doubled [11]. The cyclic tensile strain (0.5 Hz) at an amplitude of 4% or 8% promoted the alignment of TDSCs along the loading direction and the production of BMP2 [9]. In vitro, uniaxial cyclic tensile strain (4% amplitude, 0.5 Hz) promoted the alignment of TDSCs seeded in the microgrooves along the traction direction and induced their differentiation into tenocytes, while (8% amplitude, 0.5 Hz) tensile strain induced nontenocyte lineage differentiation [10]. These study results suggest that different cyclic tensile strain affects the biological properties of TDSCs. However, little work has been reported on cyclic tensile strain to promote the proliferation and tenogenic differentiation of TDSCs in three-dimensional (3D) culture in vitro.

Bioreactor can be used to simulate the 3D growing environment and natural mechanical load in vitro to promote the regeneration of functional tendon tissues [12]. A bioreactor capable of generating cyclic tensile strain at different frequencies and amplitudes has been designed and fabricated in our previous study [13–15]. Furthermore, a 3D electrospun poly(L-lactide-co-*ε*-caprolactone)/collagen (P(LLA-CL)/collagen) nanoyarn network satisfies the requirements for functional tendon tissue engineering which has been designed and fabricated in our previous study [16]. Additionally, in our previous study, we suggested that TDSCs displayed good proliferation and positive expressed tendon-related extracellular matrix (ECM) genes and proteins under cyclic tensile strain (4% amplitude, 0.5 Hz) in bioreactor culture [17]. However, the optimal mode (amplitude and frequency) of cyclic tensile strain to promote the proliferation and tenogenic differentiation of TDSCs and its underlying mechanism have not been clearly investigated.

In the present study, TDSCs were isolated from rat Achilles tendons and seeded on P(LLA-CL)/collagen scaffolds for 3D culture in the bioreactor. Our study evaluated the effect of cyclic tensile strain on the viability, proliferation, and tenogenic differentiation of rat TDSCs and revealed the most suitable cyclic tensile strain loading for tenogenic differentiation. Furthermore, the transcriptome microarray analysis was executed between cyclic tensile strain loaded TDSCs and cyclic tensile strain nonloaded TDSCs to elucidate the potential mechanism for tenogenic differentiation.

## 2. Materials and Methods

### 2.1. Mechanical Device

The mechanical device was employed for imposing cyclic tensile strain on the TDSCs-scaffold constructs. The modular bioreactor system consisted of a linear motor driver, medium circulating system, culture chamber, instrument control system, and other integrated auxiliary devices. All parameters were set according to our previous report [15]. The parameters (amplitude and frequency) of the instrument control system were adjusted to impose cyclic tensile strain on the TDSCs-scaffold constructs in the culture chamber through the linearly controlled motor driver. The stretching magnitudes (e.g., 2%) represent the axial elongation of the constructs in length.

### 2.2. Cell Culture and Preparation

**Figure 1:**
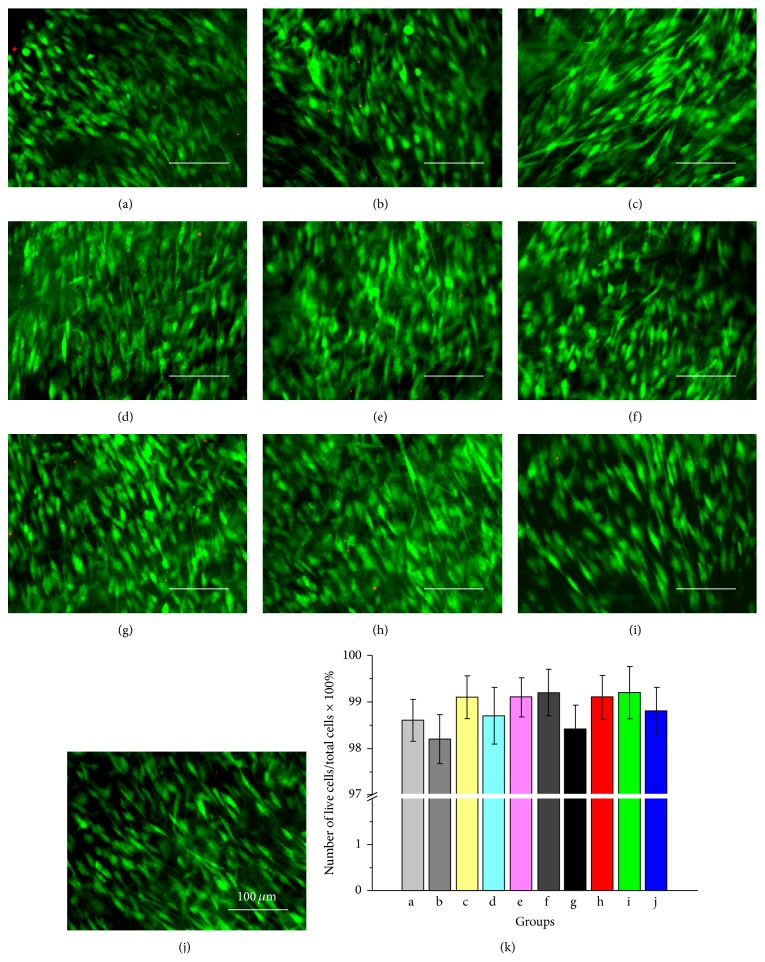
LSCM images of TDSCs on the scaffolds stained by Live/Dead assays and analysis on the proportion of the number of living cells in the total number of cells for each group. (a) Control group. (b)–(j) Experimental groups 1–9. (k) There was no statistical difference in the proportion of the number of living cells in the total number of cells between experimental groups and control group (*P* > 0.05, *n* = 3) (in all images, scale = 100 *μ*m).

Cell isolation was performed according to the procedures previously described [1, 6]. Ten male Sprague-Dawley rats (4–6 weeks old, 250–300 g, Animal Center of Daping Hospital of the Third Military Medical University) were used for all experiments. Animal experiments were approved by the Institutional Animal Care and Use Committee (IACUC) of the Third Military Medical University. All rats were sacrificed. Thereafter, their bilateral tendon calcaneus were resected, weighed, and then cut into small pieces after the tendon sheaths and the middle tendon in the paratendon were removed. 100 mg tissues were digested at 37°C for 1 h with 1 mL phosphate buffered saline (PBS) containing 3 mg type I collagenase (Sigma) and 4 mg neutral protease (Roche). The suspension was centrifuged at 500 g for 15 min. The supernatant was discarded, and the remaining cells were resuspended in Dulbecco's Modified Eagle's Medium (DMEM)/F12 (1 : 1) (HyClone) medium containing 20% fetal bovine serum (FBS; Invitrogen) and 1% penicillin and streptomycin (HyClone). The isolated cells were plated at an optimal cell density (about 500 cells/cm^2^) and cultured at 37°C and 5% CO_2_. After 8–10 days in culture, individual cell colonies were formed on the culture surface of the plate and were detached by local application of trypsin under microscopic visualization. The detached cell colonies were collected using a micropipette and mixed together as TDSCs passage 0. The cells were digested with 0.25% trypsin and passaged after reaching 90% fusion. TDSCs at passage 3 preserved good colongenicity and excellent multilineage differentiation potential, as shown by Figures 1 and 2 in Supplementary Material available online at http://dx.doi.org/10.1155/2015/790804.

### 2.3. Scaffold Preparation and Cell Seeding

P(LLA-CL)/collagen nanoyarn scaffolds were fabricated by electrospinning as described in our previous study [16]. A 3-0 nylon thread was presewn on the P(LLA-CL)/collagen scaffolds (length × width: 30 mm × 25 mm) along the broadside. The scaffold samples were placed into tissue-culture polystyrene plates (Costar) with a diameter of 10 cm and sterilized with 70% ethanol for 30 min. The scaffold samples were then rinsed five times with PBS and immersed in DMEM/F12 medium overnight. The TDSCs (passage 3) were seeded on the scaffolds (1 × 10^5^ cells/scaffold). After planting TDSCs onto the scaffolds, the cell-seeded scaffolds were placed in a 37°C, 5% CO_2_ incubator for 4 h static culture to promote cell adhesion. Then, 15 mL culture medium was added into the wells.

After the cell-seeded scaffolds were subjected to static culture for 24 h, they were curled into concentric 3D constructs along their 3 cm long axes and then fixed on the two opposing tissue fixing columns of the culture chamber by the nylon threads under sterile conditions [17]. There were totally 3 cell-scaffold constructs/culture chambers. About 80 mL culture medium was added into each culture chamber. The cell-scaffold constructs were randomly divided into experimental and control groups. Cyclic tensile strain with different parameters (amplitude and frequency) was used in the experimental groups, and the duration of cyclic tensile strain was 3 h/day, as previously reported [15]; the cell-scaffold constructs in the control group were subjected to static culture without tensile strain stimulation. The samples were cultured for 7 days, and the medium was replaced twice a week.

### 2.4. Cell Viability and Morphology

The viability and morphology of TDSCs on the scaffold samples were evaluated using Live/Dead assays (Invitrogen) in accordance with the manufacturer's instructions [18]. The samples were imaged by laser scanning confocal microscopy (LSCM; Carl Zeiss, LSM 510 META, Germany) using the excitation wavelengths of 488 nm and 594 nm. The number of viable cells and the total number of cells were counted from the images by image J software (Image J 1.46 r; National Institutes of Health), and their ratio was calculated (*n* = 3).

### 2.5. Cell Proliferation

The cell proliferation of TDSCs on the scaffold samples was determined using the 4-[3-(4-iodophenyl)-2-(4-nitrophenyl)-2H-5-tetrazolio]-1,3-benzene disulfonate (WST-1) assay (Roche, Germany) on day 1, day 3, day 5, and day 7; the samples were taken out from the culture chamber under sterile conditions, cut into pieces, and completely digested with 0.25% trypsin at 37°C. After centrifugation at 1500 rpm for 5 min [19], the supernatant was discarded and the resultant sediment was resuspended with 2 mL culture medium. According to the manufacturer's instructions, 100 *μ*L cell suspension was added into a well of the 96-well plate (10 wells/sample), and then 10 *μ*L WST-1 solution was added into each well for 3 h of incubation. The absorbance was measured at 450 nm using a microplate reader (Model 550; Bio-Rad, USA).

### 2.6. Quantitative Polymerase Chain Reaction (QPCR) Analysis

QPCR was employed to evaluate the mRNA expression levels of tendon-specific markers (type I collagen, tenascin-C, tenomodulin, and scleraxis) of TDSCs on the scaffold samples on day 7. The TDSCs collected from the control groups served as controls. GAPDH was used as housekeeping gene. The constructs were completely digested with 0.25% trypsin at 37°C. After centrifugation, the cells were collected. Total RNA was extracted from these collected cells using TRIzol Reagent (Invitrogen), and then RNA concentration was determined using a UV/Vis spectrophotometer (Thermo Fisher Scientific). cDNA was transcribed reversely using an iScript cDNA synthesis kit after DNA-erase reaction (Bio-Rad). Real-time PCR was performed with a Power SYBR Green PCR Master Mix (Applied Biosystems) on a light cycle apparatus (Applied Biosystems 7500). All primer sequences (Table 1) were designed and synthesized by Sangon Biotech Co., Ltd. (Shanghai, China). The study was repeated at least three times, and each target gene underwent three PCR cycle tests. The expression levels of target genes were calculated with 2^−ΔΔCt^ after GAPDH standardization.

### 2.7. Transcriptome Microarray Analysis

Total RNA was extracted with Qiagen RNeasy Mini Kit (Qiagen). Roche Nimblegen Rat Gene Expression array analysis was performed with Affymetrix GeneChip Rat Gene 1.0 ST Array system (Affymetrix, Santa Clara, CA). Data analysis was performed using Molecular Annotation System 3.0 (http://bioinfo.capitalbio.com/mas3/).

### 2.8. Statistical Analysis

All of the quantitative data were presented as the mean ± standard deviation. SPSS16.0 software was used for statistical analysis. Single-way analysis of variance (ANOVA) was used to assess the statistical significance of results between experimental group and control group, while two-way ANOVA was adopted to analyze the effects of different frequencies and amplitudes on the cell proliferation and the expression levels of specific genes. Differences were considered significant when *P* < 0.05.

## 3. Results

### 3.1. Cyclic Tensile Strain for Cell Viability and Proliferation

The viability and morphology of TDSCs in the control group and experimental groups cultured in the culture chamber for 7 days were shown in Figure 1. It was observed that TDSCs could maintain good viability in both the control group and experimental groups. The cells presented a long spindle shape along the scaffold fiber direction in the control group (Figure 1(a)), and TDSCs in the experimental groups also exhibited a spindle-shaped morphology along with substrate fibers and the mechanical traction direction (Figures 1(a)–1(j)). There were no significant differences in the proportion of the number of viable cells in the total number of cells between experimental groups and control group (Figure 1(k), *P* > 0.05, *n* = 3).

**Figure 2:**
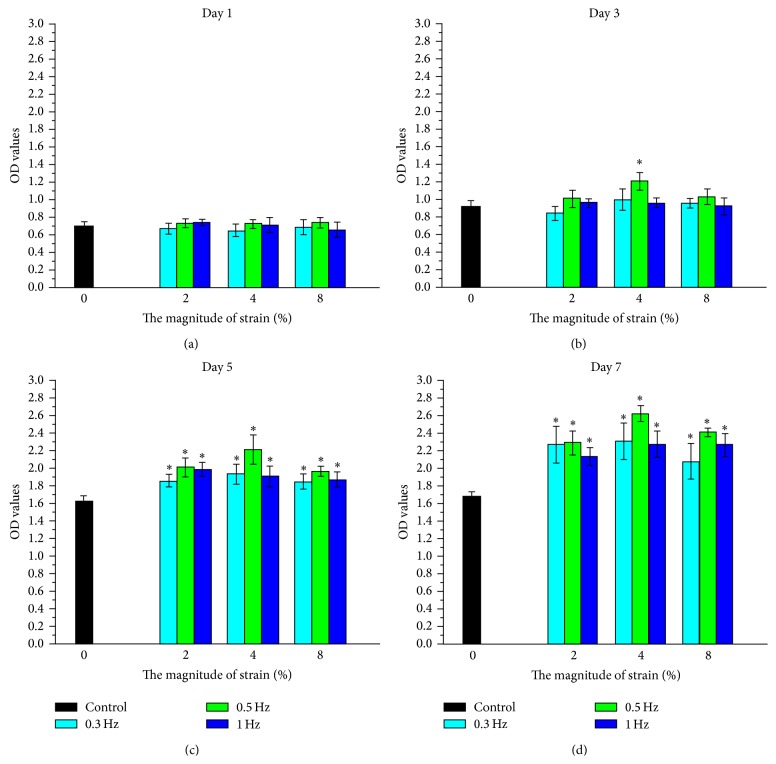
WST-1 results of TDSCs cultured in control group and different experimental groups (1 d for (a); 3 d for (b); 5 d for (c); 7 d for (d)). The data were expressed as mean ± SD. The samples indicated with asterisk (*∗*) had a significant difference between experimental groups and control group (*P* < 0.05) (*n* = 3).

The cell proliferation was measured with WST-1 assay and the results showed that (Figure 2), on day 1, there was no significant difference in cell proliferation among groups (Figure 2(a)); on day 3, significant difference in cell proliferation was found only between the 0.5 Hz/4% experimental group and the control group (*P* < 0.05, Figure 2(b)). However, there were significant differences in cell proliferation between experimental groups and control group on day 5 and day 7 (*P* < 0.05, Figures 2(c) and 2(d)), and a significant effect was observed for both strain frequencies and amplitudes on cell proliferation, and the most obvious effect was observed in 0.5 Hz experimental group at the same amplitude and in 4% experimental group at the same frequency, respectively.

### 3.2. Gene Expression of TDSCs under Cyclic Tensile Strain

**Figure 3:**
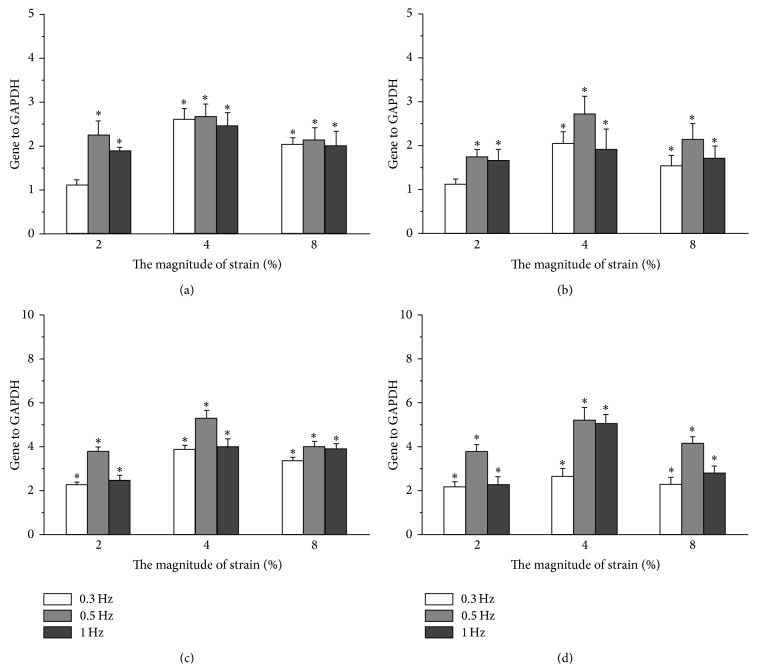
Expression of type I collagen (a), tenascin-C (b), tenomodulin (c), and scleraxis (d) of TDSCs cultured in control group and different experimental groups for 7 days. The expression levels, quantified using real-time RT-PCR, were normalized to those of housekeeping gene (GADPH). TDSCs in the control group served as controls (the expression levels of tendon ECM genes were treated as 1 in this group). The data were expressed as mean ± SD. The samples indicated with asterisk (*∗*) had a significant difference between experimental groups and control group (*P* < 0.05) (*n* = 3).

To further confirm the tendon differentiation of strain-stimulated TDSCs, we examined the tenogenic differentiation-related genes (type I collagen, tenascin-C, tenomodulin, and scleraxis) expression levels in all experimental groups after being stimulated by cyclic tensile strain and the control group (Figure 3). Compared with the control group, the significantly higher expression levels of type I collagen (Figure 3(a)) and tenascin-C (Figure 3(c)) were observed in all experimental groups except 0.3 Hz/2% experimental group after TDSCs were stimulated by cyclic tensile strain (*P* < 0.05). In addition, we also found the significantly higher expression of tenomodulin (Figure 3(c)) and scleraxis (Figure 3(d)) in all experimental groups after TDSCs were stimulated by cyclic tensile strain, as compared with the control group. Meanwhile, a significant effect was observed for both strain frequencies and amplitudes on the expression of the four tendon-specific ECM genes of TDSCs in the cell-scaffold constructs; at the same amplitude the most obvious effect was observed in 0.5 Hz experimental group, while it was seen in 4% experimental group at the same frequency, respectively.

### 3.3. Gene Expression under Optimum Cyclic Tensile Strain for Tendon Differentiation

From the above results, distinct cell proliferation and tendon differentiation of TDSCs were regulated by cyclic tensile strain. To further explore related transduction mechanisms in cellular signaling, the transcriptome differences at the mRNA level in TDSCs with cyclic tensile strain (0.5 Hz, 4% amplitude) were analyzed with Affymetrix GeneChio Rat Gene 1.0 ST transcriptome array. TDSCs cultured on same condition without cyclic tensile strain were used as controls. Each of these arrays has 27,343 well-annotated rat targets with 722,254 distinct probes based on the November 2004 rat genome sequence (UCSC rn4, Baylor HGSC build 3.4) with comprehensive coverage of RefSeq, putative complete CDS GenBank transcripts. The Rat Gene 1.0 ST Array has 99.98 percent coverage of NM sequences present in the April 3, 2007, RefSeq database.

**Figure 4:**
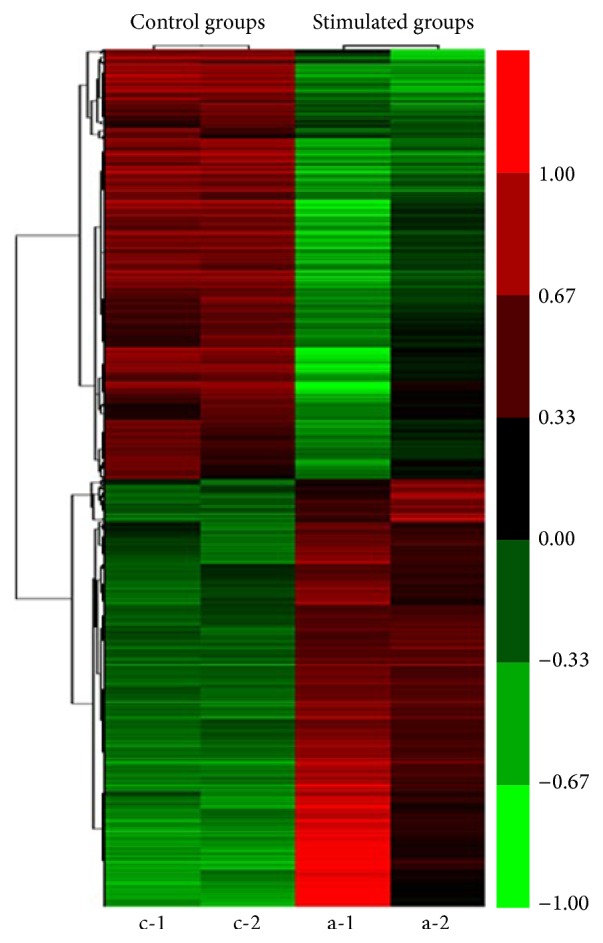
Transcriptome microarray analysis of TDSCs in the stimulated groups (a-1 and a-2) and control groups (c-1 and c-2), calculated with cluster 3.0.

Consistent expression was found in more than 30% of the transcripts with the cyclic tensile strained mRNA samples. The transcriptional clustering profiles were significantly different from no cyclic tensile strained cells (Figure 4), which was based on a threshold of *P* ≤ 0.05, and revealed that a total of 8.3% (2260 out of 27,343 transcripts) of Illumina gene sets were differentially regulated. Totally 1126 gene probe sets were upregulated and 1134 transcripts were downregulated in the cyclic tensile strained cells. Among these genes, 246 transcripts were found to be upregulated and 265 were found to be downregulated by 2-fold or more, and 36 gene products were upregulated and 83 were downregulated by 3-fold or more (*P* ≤ 0.005).

The top 20 upregulated and downregulated genes were shown in Table 2. We found that the Tubulin polymerization-promoting protein family member 3, Tppp3, ranked top in the upregulated genes. Tppp3 is a specific marker for tendon sheath differentiation [20]. And IGF-1 ranked top in the downregulated genes, which was reported to drive adipose-derived stem cells differentiating into chondrocyte-like cells [21]. Moreover, Gjb3, a glycogen cell marker, was expressed in the stem cell state, which was transiently increased during early stages of differentiation [22], and S100a10 calpactin I (S100 A10) was involved in regulating cell cycling and differentiation [23]. These data strongly indicated TDSCs differentiated into tendon cells under optimum cyclic tensile strain rather than other phenotypes such as chondrocyte-like cells.

The cyclic tensile strain-regulated genes with a change ≥2-fold (*P* ≤ 0.05) in either direction were subjected to gene ontology (GO) analysis for functional annotation with the KEGG pathways (Table 3). The upregulated genes were related to DNA replication and cell cycle and to the proteasome, which were consistent with the growth status differences. Interestingly, downregulated genes related to ECM-receptor interaction and focal adhesion [24]. Both expression levels of Lbsp and Lp1 were dropped to less than half of that in non-strain-stimulated cells, which suggested that cyclic tensile strain induces impaired ECM-receptor interaction. Moreover, IPA global functional analysis also showed regulated gene products mainly implicated in cell cycle control of chromosomal replication.

### 3.4. Signaling Network Analysis in Cyclic Tensile Strain Induced Tendon Differentiation

**Figure 5:**
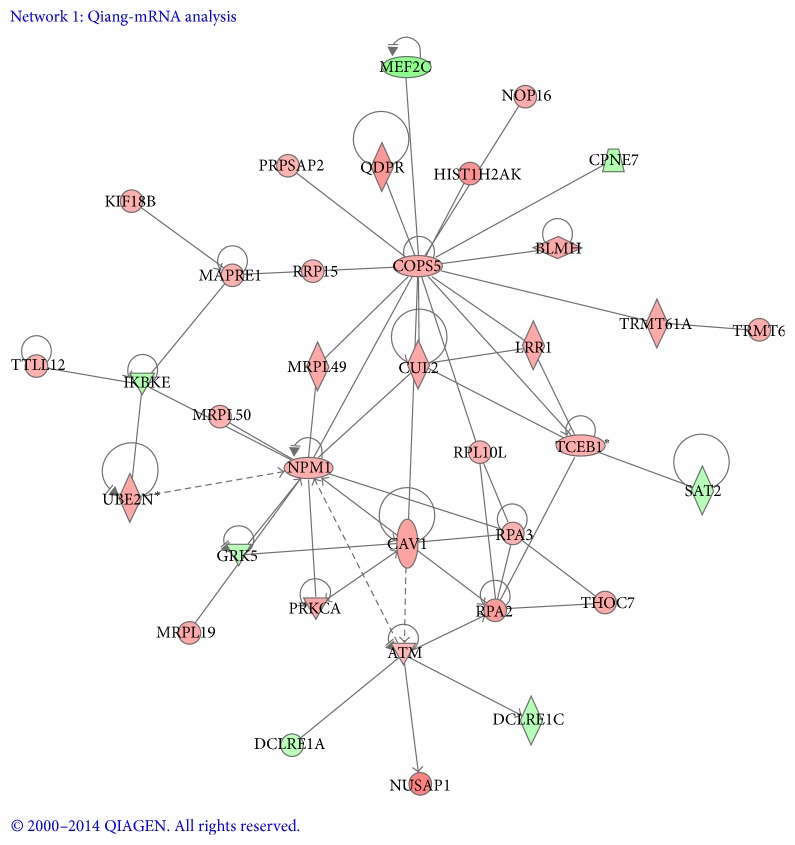
Ingenuity interactome analysis of the effect of cyclic tensile strain-affected gene expression was performed using the Ingenuity software. The gene products in network were displayed as nodes, and the biological relationships between the nodes were displayed as lines. Different shapes represented different functional classes of gene products. The color of each node indicates the degree of upregulation (red) or downregulation (green) of the respective gene transcript. Network 1: the COPS5 and NPM1 network connected with several important canonical pathways, which shows the connection of this network to DNA replication.

**Figure 6:**
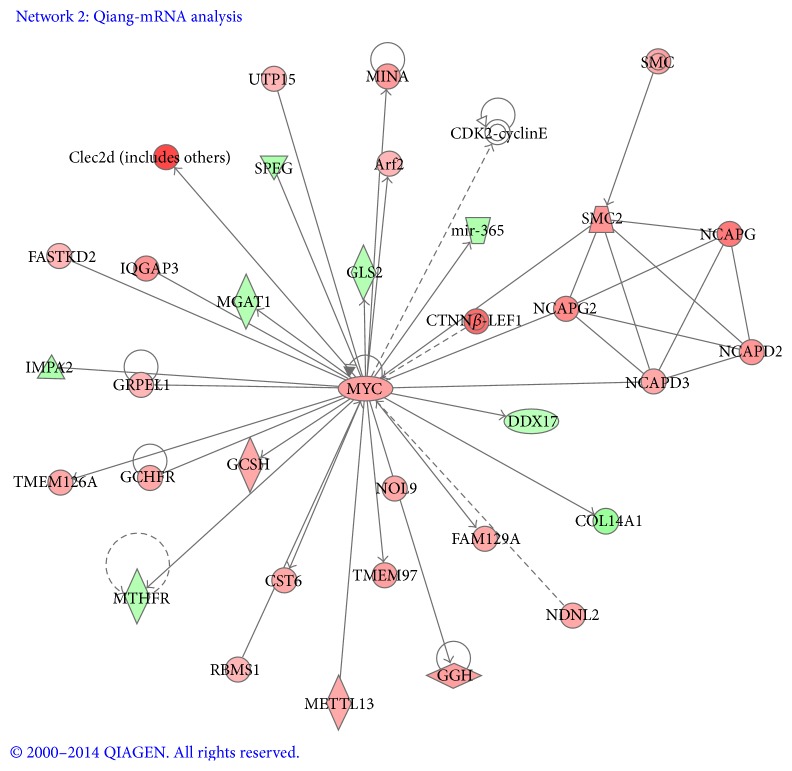
MYC signaling. MYC as a hub of connectivity appeared to play a central role possessing 30 targeted genes via direct connections.

To further understand the molecular and signaling networks for TDSCs loaded in these conditions, signaling network analysis was performed with Ingenuity Knowledge Base (IKB) and Global Molecular Network (GMN) from the Ingenuity System Database, which provided genomic interaction and signal networks information. The gene interactions and biological processes networks were analyzed by IPA program with the regulated genes with a ratio ≥2 (*P* < 0.05). IPA program gave a score for each signaling network according to the gene expression ratios, in which highest score indicated the most highly regulated network. Here, two highest scored networks with described relationships were analyzed between a series of gene subset and interacting neighboring genes in this dataset (Figures 5 and 6). Upregulated genes in the network were shown with red symbols and downregulated genes were shown with green symbols. Network 1 (Figure 5) received the highest score (44) and contains 34 significantly regulated genes. Two transcription regulators, COPS5 and NPM1, were located in the central position of this network, which connected with several important canonical pathways related to cell proliferation. NPM1 expression indicated elevated DNA replication [25]. This signaling included the significantly upregulated genes, ATM, COPS5, NPM1, RPA2, RPA3, and UBE2N, and downregulated genes, IKBKE and GRK5. The COPS5-regulated transcripts were mainly upregulated (red symbols) except for MEF2C and CPNE7 (green symbols). The NPM1 and COPS5 networks are involved in cellular and molecular functions of DNA replication, recombination and repair, cellular compromise, cell death, and survival [26].

Network 2 (Figure 6) received a score of 36 which contained 32 genes. MYC, as a hub of connectivity [27], appeared to play a central role possessing 30 edges via direct connections. Among them, GRPEL, TMEM126A, GCSH, CST6, TMEM97, NOL9, GGH, FAM129A, NDNL2, Arf2, UTP15, MINA, and Clec2d were significantly elevated, whereas MGAT1, IMPA2, MTHFR, COL14A1, DDX17, mir-365, and GLS2 were downregulated as shown. Five other transcripts belonging to the SMC (structural maintenance of chromosomes) and non-SMC condensin complex were also present as the downstream of MYC signaling [28]. All of them were upregulated. Altogether, Network 2 was in correlation with a set of cellular and molecular functions which include DNA replication, recombination and repair, cellular assembly and organization, and gene expression.

## 4. Discussion

When subjected to mechanical stimuli, viable tissues will undergo endogenous changes in cell morphology, tissue building, and mechanical properties [29, 30]. It is reported that mechanical stimulation plays an important role in the induction of cell proliferation and differentiation, cell alignment, extracellular matrix synthesis, and tissue remodeling [31, 32]. Physical and mechanical factors play a critical role in controlling self-renewal and lineage specification of the stem cells [33]. This study aimed at investigating the optimal mode (amplitude and frequency) of cyclic tensile strain to promote the proliferation and tenogenic differentiation of TDSCs and its underlying mechanism in 3D culture in vitro.

Our previous experimental study confirmed that P(LLA-CL)/collagen nanoyarn scaffolds had no effects on tenocyte adhesion and proliferation and tenocytes well spread along with aligned substrate fibers exhibiting a spindle-shaped morphology [16]. The present study also showed that TDSCs could maintain good viability on the scaffolds and grow along the scaffold fiber direction and the direction of tensile strain stimuli.

Many studies have confirmed that mechanical stimulation can promote cell proliferation, which depends on its type, magnitude, frequency, and duration. Cyclic tensile strain stimulation can promote the proliferation of anterior cruciate ligament fibroblasts, and the level of cell proliferation is higher with the increase of tensile strain amplitude (4%–8%) [34]. Obaid and Connell [8] reported that the proliferation of tendon fibroblasts induced by mechanical stimulation depended on the range of tensile strain. Cell proliferation can reflect the activity changes of cells under different modes of mechanical stimulation. In this study, cyclic tensile strain at different frequencies and amplitudes had different effects on the proliferation of TDSCs in various experimental groups. Under cyclic tensile strain, at frequencies ranging from 0.3 Hz to 1.0 Hz, the proliferation of TDSCs showed a trend of increase at first but later it showed a trend of decrease, and the most obvious stimulation effect was observed at 0.5 Hz; an identical trend was observed at amplitudes ranging from 2% to 8%, and the most obvious stimulation was found at 4%. This might be because there was a certain optimal stimulation threshold and limitation within the selected frequency or amplitude range; when the frequency or amplitude approached the threshold from the minimal value, the proliferative activity of cells was enhanced, and when the frequency or amplitude exceeded the limitation from the maximum value, the proliferative activity of cells was depressed, which is similar to the results of a recent study on the effects of fluid shear strain and fluid pressure on cell proliferation [35].

The upregulation of markers indicative of a mature, differentiated cell phenotype is the most often judgment of differentiation [36]. Type I collagen is the primary matrix components of natural tendon tissues. Type I collagen is accounting for about 85% of the dry weight of tendon tissues, and it is also the most important protein constituting tendon fibers against tensile strain. Tenascin-C is used as a tendon marker in embryonic tendon [37]. Expressions of scleraxis and tenomodulin are frequently analyzed to confirm differentiation towards a tenocyte lineage. Recent studies have shown that tensile strain (10% amplitude, 1 Hz, 2 h/day) can stimulate embryonic stem cells (ESCs) to express tendon-related genes, type I and III collagen, Epha 4, and scleraxis [38], and cyclic tensile strain can promote the tenogenic differentiation of mesenchymal stem cells (MSCs) and the repair of injury tendon [39, 40]. Our study results showed higher expression levels of tenogenic differentiation-related mRNA when TDSCs were stimulated by cyclic tensile strain. These gene expression changes were correlated with the amplitude and frequency of cyclic tensile strain. Real-time PCR results showed that there was a significant increase in the expression levels of tenogenic differentiation-related mRNA when TDSCs were stimulated by cyclic tensile strain in all experiments. At the same amplitude, the stimulation effects of strain on the expression of type I collagen, tenascin-C, tenomodulin, and scleraxis showed frequency-dependent increasing and then decreasing trend at different frequencies, wherein the effect at 0.5 Hz was the strongest; at the same frequency, the stimulation effects of strain on the expression of type I collagen, tenascin-C, tenomodulin, and scleraxis showed amplitude-dependent increasing and then decreasing trend at different amplitudes, wherein the effect at 4% was the strongest. The frequency and amplitude of cyclic tensile strain imposed have no superimposing or offsetting effects on the expression levels of these four genes. Microscopic tearing of tendon fibers occurs when tendons are stretched over 4% and macroscopic failure occurs when tendons are stretched beyond 8–10% strain [41]. Our results showed that, at various frequencies/amplitudes, cyclic tensile strain could induce the expression of tenogenic differentiation-related genes of TDSCs; the amplitude of 4% and the frequency of 0.5 Hz might be the optimal induction condition.

Results from transcriptome microarray further confirmed that cyclic tensile strain induced TDSCs had distinct cell proliferation signaling, which can explain better cell viability and proliferation with cyclic tensile strain treatment. More importantly, cell/material interface has been shown to exert considerable influence on function and differentiation of TDSC. ECM-receptor signaling played a crucial role in this process [24]. Impaired  ECM-receptor interaction signaling pathway was also found when treated with cyclic tensile strain, which means cyclic tensile strain can inhibit this signal to trigger TDSC differentiation. Therefore, reduced ECM receptor interaction signaling may initiate tendon genius switch.

We showed that cyclic tensile strain highly upregulates genes encoding regulators of NPM1 and COPS5 transcriptional activities as well as MYC related transcriptional factors. The networks are mainly contributed to cellular and molecular functions of DNA replication, recombination and repair, cellular compromise, cell death and survival, cellular assembly and organization, and gene expression [25–28], which also played a crucial role for tendon differentiation and cell proliferation.

In addition, one limitation of this study is that the amplitude and frequency as well as duration ranges of cyclic tensile strain are not detailed enough, so we will perform further screening. Besides, the cyclic tensile strain we used may be different from the composite mechanical stimulation withstood by natural tendon tissues in vivo; thus we will further improve our mechanical force devices to provide multiple modes of mechanical stimulation.

## 5. Conclusions

The custom-designed 3D tensile bioreactor used in our study provides testimonies that cyclic tensile strain with different parameters has different effects on the proliferation and tenogenic differentiation of TDSCs; cyclic tensile strain with 0.5 Hz at 4% amplitude may be the optimal condition for the proliferation and tenogenic differentiation of TDSCs. Moreover, the transcriptome analysis provided certain new insights on the molecular and signaling networks for TDSCs loaded in these conditions.

## Supplementary Material

Total TDSCs colonies stained with Methyl violet, TDSCs at passage 3 preserved good colongenicity, as shown by Fig. 1 in the Supplementary data. The multi-differentiationpotential of the TDSCs was tested in vitro for adipogenesis, chondrogenesis, and osteogenesis, TDSCs at passage 3 were also proved with excellent multi-lineage differentiation potential, as shown by Fig. 2 in the Supplementary data.

## Figures and Tables

**Table 1:** Real-time PCR primers used in this study. Primers for type I collagen, tenascin-C, tenomodulin, and scleraxis and GAPDH were designed and synthesized by Sangon Biotech Co., Ltd.

Gene	Sequence	
Type I collagen	Forward	5′ tcagaacatcacctaccactgc 3′
	Reverse	5′ attgtctttccccattcatttg 3′

Tenascin-C	Forward	5′ atcaccaccaagttcacaacag 3′
	Reverse	5′ ccatccacagattcatagagca 3′

Tenomodulin	Forward	5′ tccacaattcggcataatc 3′
	Reverse	5′ caggtccgggattctgtgt 3′

Scleraxis	Forward	5′ ccacaccaagcattttcaga 3′
	Reverse	5′acacaaaggacggcatcac 3′

GAPDH	Forward	5′ atggtgaaggtcggagtgaa 3′
	Reverse	5′ tgggtggaatcatactggaac 3′

**Table 2:** The top 20 upregulated and downregulated genes with cyclic tensile strain or not.

Fold change	Gene symbol	Gene description
9.32	Tppp3	Tubulin polymerization-promoting protein family member 3
5.38	Rrad	Ras-related associated with diabetes
5.31	Aass	Aminoadipate-semialdehyde synthase
5.2	Elmod1	ELMO/CED-12 domain containing 1
4.88	Crabp2	Cellular retinoic acid binding protein 2
4.65	S100a5	S100 calcium binding protein A5
4.44	Gjb3	Gap junction protein, beta 3
4.2	Kcnn4	Potassium intermediate/small conductance calcium-activated channel, subfamily N, member 4
3.98	Mthfs	5,10-Methenyltetrahydrofolate synthetase (5-formyltetrahydrofolate cyclo-ligase)
3.97	Lmo1	LIM domain only 1
3.83	Cxcl10	Chemokine (C-X-C motif) ligand 10
3.8	S100a10	S100 calcium binding protein A10
3.78	Anxa8	Annexin A8
3.73	Tspan2	Tetraspanin 2
3.68	Ezr	Ezrin
3.66	Crip1	Cysteine-rich protein 1 (intestinal)
3.58	Mlf1ip	Myeloid leukemia factor 1 interacting protein
3.54	Clec2dl1	C-type lectin domain family 2 member D-like 1
3.53	Fam111a	Family with sequence similarity 111, member A
3.38	Atp2b4	ATPase, Ca++ transporting, plasma membrane 4

0.12	IGF-1	Insulin-like growth factor 1
0.14	Stc1	Stanniocalcin 1
0.16	Olr1	Oxidized low density lipoprotein (lectin-like) receptor 1
0.17	Nkd2	Naked cuticle homolog 2 (*Drosophila*)
0.18	Lpl	Lipoprotein lipase
0.18	Rgs16	Regulator of G-protein signaling 16
0.19	LOC24906	RoBo-1
0.19	Slc15a3	Solute carrier family 15, member 3
0.2	Kbtbd10	Kelch repeat and BTB (POZ) domain containing 10
0.21	Rgs2	Regulator of G-protein signaling 2
0.22	RGD1308023	Similar to CG5521-PA
0.23	RGD1308023	Similar to CG5521-PA
0.23	RGD1308023	Similar to CG5521-PA
0.24	Bmp4	Bone morphogenetic protein 4
0.24	Ibsp	Integrin-binding sialoprotein
0.24	Kcnj2	Potassium inwardly rectifying channel, subfamily J, member 2
0.24	Lmcd1	LIM and cysteine-rich domains 1
0.25	Agtr1a	Angiotensin II receptor, type 1a
0.25	Avpr1a	Arginine vasopressin receptor 1A
0.25	Fat4	FAT tumor suppressor homolog 4 (*Drosophila*)

**Table 3:** KEGG signaling pathways associated with transcriptome expression differences between the cyclic tensile strain and no treatment.

Term	Count	%	*P* value	Genes
rno03030: DNA replication	21	2.341137124	1.00*E* − 17	LIG1, POLE, MCM2, POLA2, MCM3, LOC317415, RNASEH2B, MCM4, MCM5, MCM6, RPA3, PRIM1, RFC5, RPA2, MCM7, RFC4, RFC2, POLE3, POLD2, PRIM2, PCNA, FEN1

rno04110: cell cycle	34	3.790412486	9.83*E* − 16	E2F1, E2F5, DBF4, TTK, CHEK1, CHEK2, CCNE2, CCNE1, CDKN2A, MCM7, BUB1, MYC, CCNA2, BUB3, CDC6, RBL1, ESPL1, CDC20, CDK6, MCM2, CDC25C, MCM3, LOC317415, MCM4, MCM5, ATM, SMC3, MCM6, CCNB1, CCND1, MAD2L1, CCNB2, PLK1, PCNA, BUB1B

rno03050: proteasome	18	2.006688963	1.27*E* − 10	PSMB10, PSMB4, PSMB7, PSMC6, PSMD14, PSMB6, PSMC5, PSMA6, PSMC4, PSMB1, PSMA5, PSME2, PSMA4, PSMB3, PSMC1, PSMA3, POMP, PSMD6

rno00240: pyrimidine metabolism	20	2.229654404	1.06*E* − 07	POLR3G, POLR1E, CTPS, POLE, POLR1A, POLR1C, CAD, POLA2, POLR3D, PRIM1, TYMS, UMPS, NME2, POLE3, NT5C3, RRM2, POLD2, PRIM2, UCK2, DUT

rno03430: mismatch repair	10	1.114827202	5.35*E* − 07	EXO1, RFC5, RPA2, RFC4, RFC2, MSH2, LIG1, POLD2, PCNA, RPA3

rno04115: p53 signaling pathway	15	1.672240803	3.77*E* − 06	BID, STEAP3, CDK6, CHEK1, CHEK2, ATM, GTSE1, CCNE2, CCNB1, CCNE1, CCND1, CASP3, CDKN2A, CCNB2, RRM2

rno03420: nucleotide excision repair	11	1.226309922	4.08*E* − 05	RFC5, RPA2, RFC4, POLE3, RFC2, LIG1, POLE, POLD2, PCNA, ERCC1, RPA3

rno00230: purine metabolism	21	2.341137124	8.38*E* − 05	POLR3G, POLR1E, NUDT5, POLE, POLR1A, POLR1C, POLA2, PPAT, GART, POLR3D, PRIM1, NME2, POLE3, ATIC, NT5C3, RRM2, ADK, POLD2, PRIM2, PRPS2, PRPS1

rno00670: one carbon pool by folate	6	0.668896321	6.33*E* − 04	MTHFD1, MTHFS, TYMS, DHFR, ATIC, GART

rno03440: homologous recombination	7	0.780379041	0.001556012	RPA2, NBN, POLD2, BRCA2, RAD54L, RPA3, RAD51

rno03410: base excision repair	8	0.891861761	0.002577441	POLE3, NEIL3, LIG1, POLE, POLD2, PCNA, APEX1, FEN1

rno04114: oocyte meiosis	14	1.560758082	0.003751587	PPP2R1B, SGOL1, ESPL1, CDC20, CDC25C, SMC3, CCNB1, CCNE2, CCNE1, MAD2L1, CCNB2, PLK1, BUB1, FBXO5

rno00270: cysteine and methionine metabolism	7	0.780379041	0.00748193	GOT1, AHCY, DNMT1, AHCYL2, AMD1, APIP, SMS

rno05322: systemic lupus erythematosus	11	1.226309922	0.014926373	HIST1H2BA, HIST1H2BB, HIST1H2BH, SSB, LOC682330, LOC680498, HIST1H2BM, HIST1H4B, HIST2H2AC, HIST1H2AI, HIST3H2A, H2AFX, HIST1H2AO

rno03040: spliceosome	13	1.449275362	0.018817601	PRPF31, PPIL1, EFTUD2, MAGOH, SNRPB2, CWC15, SNRPD2, SF3B2, PRPF19, PLRG1, THOC4, LSM3, SNRPF

rno00480: glutathione metabolism	7	0.780379041	0.039033496	LAP3, ODC1, GSTA2, GSTA4, RRM2, SMS, GCLM

rno03020: RNA polymerase	5	0.557413601	0.039888482	POLR3G, POLR1E, POLR1A, POLR1C, POLR3D

rno04512: ECM-receptor interaction	16	2.285714286	7.51*E* − 07	IBSP, COL4A2, ITGA1, ITGA11, LAMA2, LAMA4, LAMB2, LAMA5, ITGB8, ITGA7, TNN, AGRN, LAMC1, COL11A1, THBS2, THBS4

rno04510: focal adhesion	24	3.428571429	3.96*E* − 06	IBSP, COL4A2, VAV3, ITGA11, ITGA1, IGF1, HGF, MYL9, LAMA2, LAMA4, LAMB2, LAMA5, ITGB8, ITGA7, PDGFRA, PDGFRB, TNN, LAMC1, EGF, COL11A1, FIGF, THBS2, SHC4, THBS4

rno05414: dilated cardiomyopathy	11	1.571428571	0.003630871	LAMA2, ADCY4, ITGB8, ITGA7, ADCY6, ITGA11, ITGA1, IGF1, CACNB3, CACNA1C, SGCB

rno04270: vascular smooth muscle contraction	12	1.714285714	0.00645965	RAMP3, ADCY4, AGTR1A, ADORA2A, PPP1R12B, ADCY6, AVPR1A, NPR2, PRKG1, CACNA1C, PRKCE, MYL9

rno00230: purine metabolism	14	2	0.010450898	ADCY4, ENPP3, ADCY6, PDE10A, PDE3A, NPR2, PDE4D, AMPD3, POLD4, PDE7B, PDE4B, ENTPD4, ENTPD1, ENTPD2

rno05410: hypertrophic cardiomyopathy (HCM)	9	1.285714286	0.02148213	LAMA2, ITGB8, ITGA7, ITGA11, ITGA1, IGF1, CACNB3, CACNA1C, SGCB

rno05412: arrhythmogenic right ventricular cardiomyopathy (ARVC)	8	1.142857143	0.031599478	LAMA2, ITGB8, ITGA7, ITGA11, ITGA1, CACNB3, CACNA1C, SGCB

rno04614: renin-angiotensin system	4	0.571428571	0.046740741	AGTR1A, MME, ANPEP, ENPEP
