# Supplementary material for: Cyclic Tensile Strain Induces Tenogenic Differentiation of Tendon-Derived Stem Cells in Bioreactor Culture
Source: Biomed Res Int. 2015 Jul 1;2015:790804. doi: 10.1155/2015/790804 (PMC4502284; doi:10.1155/2015/790804)
Supplement: Supplementary file 1 — Total TDSCs colonies stained with Methyl violet, TDSCs at passage 3 preserved good colongenicity, as shown by Fig. 1 in the Supplementary data. The multi-differentiationpotential of the TDSCs was tested in vitro for adipogenesis, chondrogenesis, and osteogenesis, TDSCs at passage 3 were also proved with excellent multi-lineage differentiation potential, as shown by Fig. 2 in the Supplementary data. [file 790804.f1.docx]

**Supplementary data**


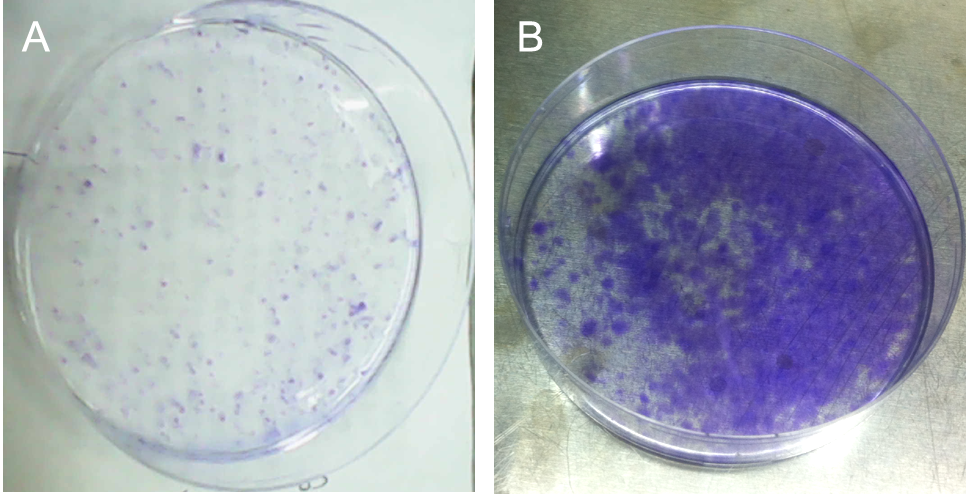


*Fig.1. The colony formation of rabbit tendon stem cells (TDSCs). Total TDSCs colonies stained with Methyl violet at passage 3 cultured in 10 cm Petri dish.*

*A: at day 2. B:at day 5.*


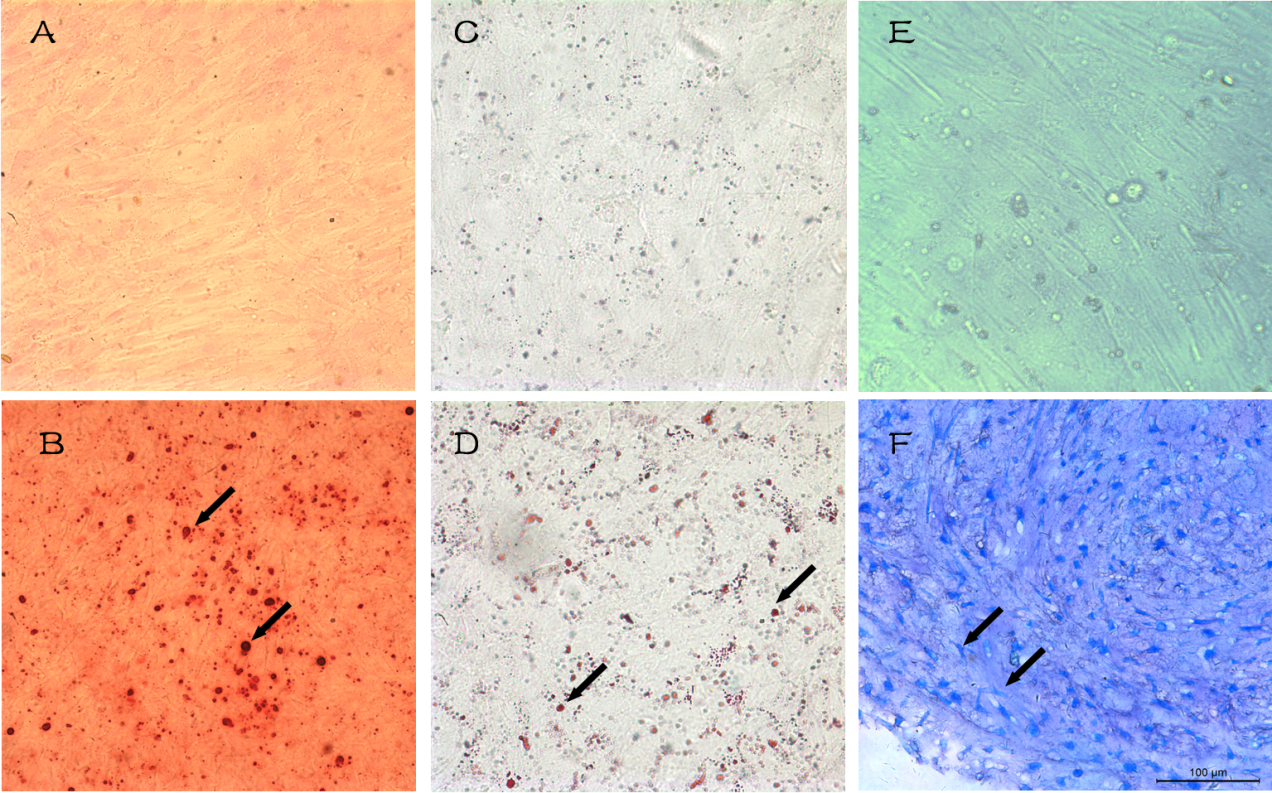


*Fig.2. The testing of multi-differentiation potential of TDSCs in vitro. A. Osteogenesis of TDSCs (Alizarin red S assay, arrows point to clustered calcium droplet).C Adipogenesis of TDSCs (Oil red O assay, arrows point to lipid droplets).E Chondrogenesis of TDSCs.(Toluidine blue assay, Blue metachromatic granules containing a large amount of GAG were seen in TDSCs (arrows)). TDSCs cultured in the control groups were not found to exhibit such a multi-differentiation potential (B,D,F).The scale on all images =100µm.*
